# Equipment-related critical incidents in a general intensive care unit

**DOI:** 10.1186/2197-425X-3-S1-A70

**Published:** 2015-10-01

**Authors:** AT King, R Wenstone, J Morrison, L Cloherty, ID Welters

**Affiliations:** Intensive Care Unit, Royal Liverpool University Hospital, Liverpool, United Kingdom; Institute of Ageing and Chronic Disease, University of Liverpool, Liverpool, United Kingdom

## Introduction

The complexity of the care provided for critically ill patients frequently leads to medical errors with potential harm. To improve standards of care, each incident should be recorded, reported and discussed within the team. The reporting system highlights where errors were made and what lessons can be learnt from such incidents. These are commonly reported as “critical incidents” or “serious incidents”. A study by Welters et al (2011) found equipment errors accounted for a large number of critical incidents (CI) [1]. It has been found that CIs may lead to longer hospital stays, increasing the cost of patient care; therefore further research into CIs and their prevention is justified [2].

## Objectives

This report investigates the incidence of equipment-related incidents within an ICU of a large inner-city hospital. In order to identify the most common equipment related CIs.

## Methods

The data included in this report was collected from a 17 bed ICU of a large inner city hospital, in the North West of England. The data was collected from 2002-2015. Each CI related to equipment was recorded, and source and mechanism of the error were identified.

## Results

Between July 2002 and February 2015, 564 CIs related to equipment were reported. There were 94 different items of equipment involved in these CIs. Interestingly, CIs relating to use of beds, mattresses and chairs were most common, with 70 (12.41%) CIs. The other most common pieces of equipment include intravenous infusion pumps (68, 12.05%), tracheostomies (51, 9.04%), ventilators (39, 6.91%), central lines (32, 6.67%), chest drains (20, 3.55%), invasive monitoring (16, 2.84%), renal replacement therapy (15, 2.66%), bronchoscopy (14, 2.48%), giving sets (11, 1.95%), blood gas analysers (11, 1.95%), and CPAP (11, 1.95%).

## Conclusions

CI reporting has an important role in any healthcare setting. It allows staff to acknowledge where errors were made, and provides an opportunity to learn from these incidents. By identifying the major sources of equipment-related incidents, appropriate training schemes can be developed to avoid such incidents in the future in order to improve patient safety.

## References

[1] Welters ID, Gibson J, Mogk M, Wenstone R (2011). Major sources of critical incidents in intensive care. Crit Care.

[2] Kaushal R, Bates DW, Franz C, Soukup JR, Rothschild JM (2007). Costs of adverse events in critical care units. Crit Care Med.

